# Logistic regression analysis of risk factors for pediatric burns: a case–control study in underdeveloped minority areas in China

**DOI:** 10.3389/fped.2024.1365492

**Published:** 2024-04-09

**Authors:** Ziren Lin, Petchi Iyappan, Zhiqun Huang, Suren Rao Sooranna, Yongfang Wu, Liuting Lan, Cheng Huang, Feiteng Liang, Daji Zhao, Dingjin Huang

**Affiliations:** ^1^ Department of Burn Surgery, The People’s Hospital of Baise, Baise, Guangxi, China; ^2^ Faculty of Pharmacy and Biomedical Sciences, MAHSA University, Kuala Lumpur, Malaysia; ^3^ Department of Burn Surgery, Affiliated Hospital of Youjiang Medical University for Nationalities, Baise, Guangxi, China; ^4^ Department of Metabolism, Digestion and Reproduction, Imperial College London, Chelsea and Westminster Hospital, London, United Kingdom; ^5^ Life Science and Clinical Research Center, Youjiang Medical University for Nationalities, Baise, Guangxi, China; ^6^ Department of Pediatrics, The People’s Hospital of Baise, Baise, Guangxi, China

**Keywords:** burns, children, risk factors, logistic regression analysis, minority, underdeveloped area

## Abstract

**Background:**

Pediatric burns are common, especially in underdeveloped countries, and these can physically affect the children involved and have an impact on their mental health. The aim of the present study was to assess the effect of pediatric burns in underdeveloped minority areas of China.

**Methods:**

Case information from 192 children was collected from outpatient and inpatient clinics using a survey questionnaire. These included 90 pediatric burn cases and 102 controls who were children without burns. A stepwise logistic regression analysis was used to determine the risk factors for pediatric burns in order to establish a model. The goodness-of-fit for the model was assessed using the Hosmer and Lemeshow test as well as receiver operating characteristic and internal calibration curves. A nomogram was then used to analyze the contribution of each influencing factor to the pediatric burns model.

**Results:**

Seven variables, including gender, age, ethnic minority, the household register, mother’s employment status, mother’s education and number of children, were analyzed for both groups of children. Of these, age, ethnic minority, mother’s employment status and number of children in a household were found to be related to the occurrence of pediatric burns using univariate logistic regression analysis (*p* < 0.05). After a collinearity diagnosis, a multivariate logistic regression analysis of variables with tolerances of >0.2 and variance inflation factor <5 showed that age was a protective factor for pediatric burns [odds ratio (OR)* *=* *0.725; 95% confidence interval (CI): 0.665–0.801]. Compared with single-child parents, those with two children were at greater risk of pediatric burns (OR* *=* *0.389; 95% CI: 0.158–0.959). The ethnic minority of the child and the mother’s employment status were also risk factors (OR* *=* *6.793; 95% CI: 2.203–20.946 and OR* *=* *2.266; 95% CI: 1.025–5.012, respectively). Evaluation of the model used was found to be stable. A nomogram showed that the contribution in the children burns model was age* *>* *mother’s employment status* *>* *number of children* *>* *ethnic minority.

**Conclusions:**

This study showed that there are several risk factors strongly correlated to pediatric burns, including age, ethnic minority, the number of children in a household and mother’s employment status. Government officials should direct their preventive approach to tackling the problem of pediatric burns by promoting awareness of these findings.

## Introduction

Burns are the fifth leading contributor of childhood injuries worldwide (1). Pediatric burns can not only affect the physical health of children, but they can also have a huge impact on their mental health. Approximately 500,000 children are reported to require hospitalization for burns worldwide annually, and it is noteworthy that most of these cases occur in developing countries (2). However, every area has its unique different socioeconomic development with different living habits and several factors can influence pediatric burns. Therefore, it is difficult to reduce and prevent these incidents that can vary even in a single country, such as China, where the incidence of pediatric burns varies in different geographical regions (3). At present, there are studies about the factors related to burns in ethnic minorities and underdeveloped areas. Due to the differences in regional economic development, social environment, living habits, and other factors, the associated factors leading to pediatric burns can also be different. Therefore, our team used a case–control study to analyze the risk factors of pediatric burns in Baise, Guangxi, China, with the aim of reducing the incidence of unnecessary hardships to the population.

## Methods

### Study population

Pediatric patients aged under 12 years who visited the Department of Burn Surgery and Department of Pediatrics, the People's Hospital of Baise between 1 November 2021 and 31 August 2023 met the inclusion criteria for the study. All the children were identified as based in Baise City according to their household registration certificate. The children were divided into two groups. The burn group (*n* = 90) consisted of children with accidental burns caused by various causes, and their condition was stable. Of the 90 cases of pediatric burns, 64 were due to scalding, 20 were due to flame burns, 2 were contact burns, 2 were chemical burns, 1 was an electrical burn, and 1 was the result of an explosion. A total of 73 cases of pediatric burns occurred at home and the data revealed that burns most commonly took place in the bathroom and living room (60 cases) followed by the kitchen (13 cases). There were 14 cases of pediatric burns that occurred outdoors, and 3 cases were grouped as “other” as these were unspecified. All pediatric burns were examined within 6 months after the burn event. The control group (*n* = 102) included children without burns who had no other diseases and their condition was stable. These patients visited the Emergency and Pediatric Departments for various reasons, including respiratory infections, abdominal pain, emesis, fever, and general child health examinations. The parents and guardians of all the participants volunteered to participate in the questionnaire and cooperated with completing the records.

Pediatric patients aged 12 years and older were excluded. In addition, some patients met the enrollment requirements, but their condition was unstable and therefore they were excluded. Those whose parents or guardians did not agree to cooperate with the questionnaire survey were also excluded.

### Statement of ethics

This study was approved by the Ethics Committee of the People's Hospital of Baise (Ethics No.: KY2023121801). All parents/guardians of participants signed an informed consent form. All research procedures involving human participants complied with the Declaration of Helsinki of 1964 and its subsequent amendments or similar ethical standards (4). Our research project used a questionnaire survey and the respondents with difficulties were subjected to face-to-face interviews.

### Data analysis and statistics

A stepwise regression analysis was performed based on the demographic data and related factors affecting the onset of children who had burns. The data were subjected to binary, univariate, and multivariate logistic regression analyses to determine the risk factors of pediatric burns using SPSS 26.0 statistical software. *p *<* *0.05 was considered to be statistically significant. The risk factors of pediatric burns were first determined and assigned to meet the needs of the binary logistic regression analysis (Table 1).

**Table 1:** Variables and assignments of pediatric burns.

Covariate	Assignment
Gender	Girl = 1, boy = 2
Age (years)	Actual age assignment
Household register	Urban = 1, rural = 2
Minority	No = 1, yes = 2
Mother education	High school or above = 1
	Junior high school or below = 2
Mother working status	Employed = 1
	Farmer = 2
	Unemployed or migrant worker = 3
Number of children	One = 1, two = 2, ≥three = 3
Dependent variable: burns	No = 0, yes = 1

Covariations in univariate logistic regression analysis were tested for multicollinearity and then a multivariate logistic regression analysis was performed. If either the tolerance (Tol) was less than 0.1 or the variance inflation factor (VIF) was greater than 5, then collinearity was assessed as present. The goodness-of-fit of the model was assessed using the Hosmer and Lemeshow test, area under the receiver operating characteristic (ROC) curve (AUC), and the internal calibration curve (ICC).

Regarding the Hosmer and Lemeshow test, model goodness-of-fit was assessed to be not good if *p *<* *0.05. Regarding the AUC, the AUC was the area below the ROC curve and represented a composite index of model performance. The AUC value was in the range of 0–1, where a value closer to 1 indicated a better model performance, and an AUC of 0.5 indicated a performance equivalent to random guessing. Generally, the closer the AUC is to 0.5, the lower the model prediction. The ICC is an important tool in evaluating prediction models, especially for calibrating the accuracy of model predictions. The resulting reliability of the nomogram was assessed by the correspondence between the predicted and the actual probabilities. If the C-index trend was closer to 1, this indicated the reliability of the results of the nomogram analysis (mean absolute error* *<* *0.05). After performing the logistic regression modeling, the coefficient of the covariate was analyzed using a nomogram (coefficient plot) in R Studio 2023.06.0.

## Results

### Demographic data

**Figure 1:**
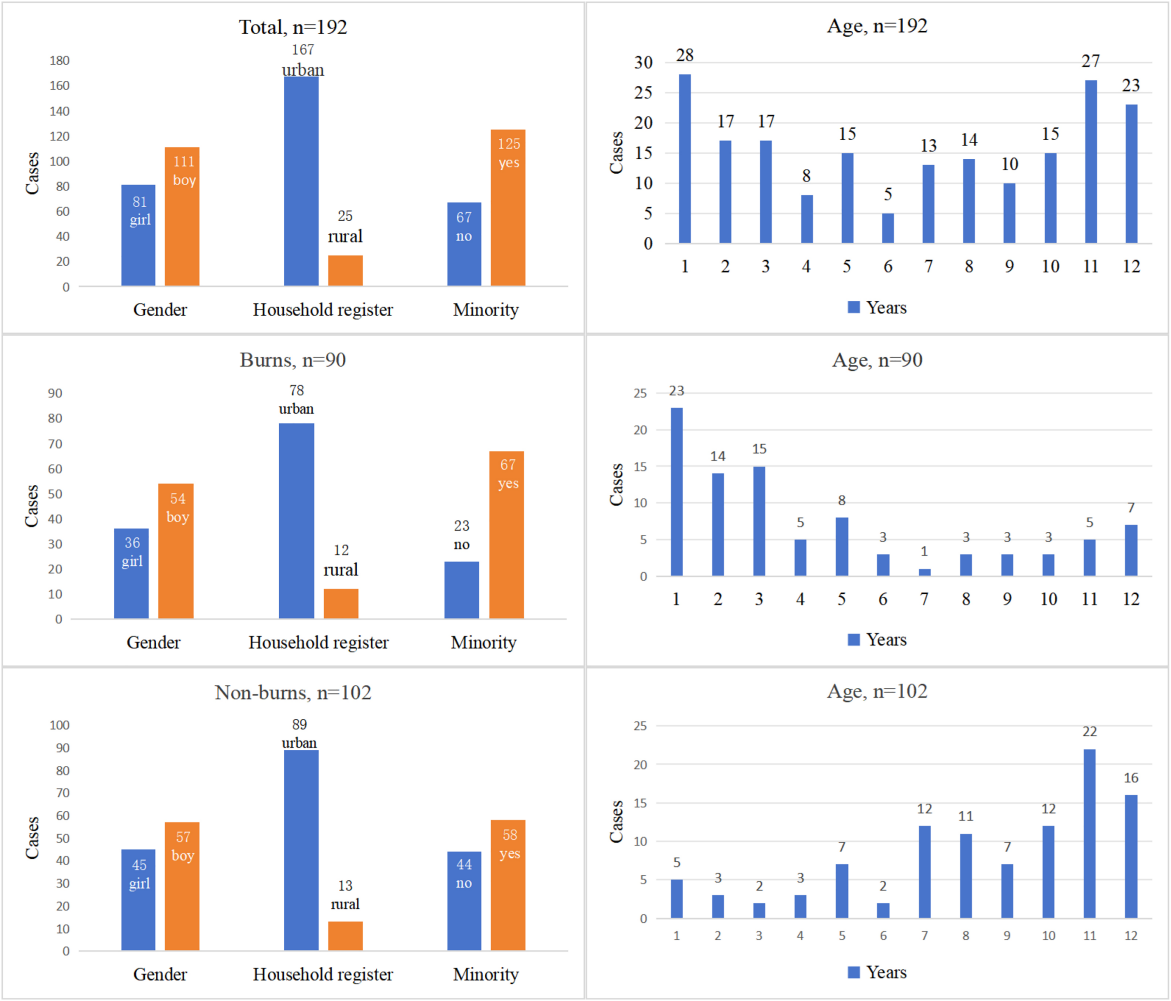
Epidemiological characteristics of the hospitalized children.

A total of 192 children were included in this study, including 90 children with burns in the case group and 102 children without burns in the control group. Of the patients, 111 were boys and 81 were girls, with a mean age of 6.59 ± 3.97 years. There were 54 boys and 36 girls in the case group, with a mean age of 4.46 ± 3.69 years. The control group consisted of 57 boys and 45 girls, with a mean age of 8.48 ± 3.18 years. The children with burns accounted for 90/192 (46.88%) patients (Table 2, Figure 1).

**Table 2:** Epidemiological characteristics of the hospitalized children.

Category	Total (*n* = 192) *N* (%) or mean ± SD	Burns (*n* = 90) *N* (%) or mean ± SD	Non-burns (*n* = 102) *N* (%) or mean ± SD
Gender			
Boy	111 (57.81)	54 (60.00)	57 (55.88)
Girl	81 (42.19)	36 (40.00)	45 (44.12)
Age (years)	6.59 ± 3.97	4.46 ± 3.69	8.48 ± 3.18
Minority			
Yes	125 (65.10)	67 (74.44)	58 (56.86)
No	67 (34.90)	23 (25.56)	44 (43.14)
Household register			
Urban	25 (13.02)	12 (13.33)	13 (12.75)
Rural	167 (86.98)	78 (86.67)	89 (87.25)

SD, standard deviation.

### Univariate logistic regression analyses of pediatric burns

Gender, age, ethnic minority, household register, mother’s education, mother’s employment status, and the number of siblings were analyzed by univariate logistic regression analysis. The results showed that age, ethnic minority, mother’s employment status, and the number of children within a family were related to the occurrence of pediatric burns, and the difference between the two groups was statistically significant (*p *<* *0.05) (Table 3).

**Table 3:** The results of univariate logistic regression analyses of pediatric burns.

Category	*β*	SE	Wald	*p*-value	OR	95% CI for OR	
						Lower	Upper
Gender							
Girl							
Boy	0.169	0.293	0.332	0.564	1.184	0.666	2.104
Age (years)	−0.299	0.046	42.109	0.000	0.742	0.678	0.812
Household register							
Urban							
Rural	−0.052	0.429	0.015	0.904	0.949	0.409	2.202
Ethnic minority							
No							
Yes	0.793	0.314	6.392	0.011	2.210	1.195	4.086
Mothers’ working status							
Employed			6.559	0.038			
Farmer	0.971	0.447	4.726	0.030	2.640	1.100	6.333
Unemployed or migrant worker	1.016	0.409	6.162	0.013	2.762	1.238	6.160
Mothers’ education							
High school or above							
Junior high school or below	0.274	0.319	0.739	0.390	1.315	0.704	2.457
Number of siblings							
One			9.996	0.007			
Two	−1.012	0.370	7.472	0.006	0.364	0.176	0.751
≥Three	−1.237	0.426	8.424	0.004	0.290	0.126	0.669

### Collinearity diagnostics

In the regression analysis, because each variable was not isolated, there may have been some dependence on each other. However, this relationship can increase the standard error, mean square error of the parameters, and the opposite direction of the regression coefficient, and these can result in the unreasonable fitting of the logistic regression model. Therefore, we used a collinearity diagnosis of the factors that were statistically significant in univariate logistic regression analyses. These were age, mother’s employment status, ethnic minority, and the number of children in the family. The covariate with *s* tolerance* *>* *0.2 and VIF* *<* *5 was then subjected to the multivariate logistic regression analysis (Table 4).

**Table 4:** Collinearity diagnostics results for age, mothers’ working status, ethnic minority, and number of children.

Category	Tolerance	VIF
Age	0.957	1.045
Ethnic minority	0.936	1.068
Number of children in a family	0.896	1.116
Mothers’ working status	0.974	1.027

### Multivariable logistic regression analyses of pediatric burns

The results of the multivariable regression analyses showed that age was a protective factor for pediatric burns [odds ratio (OR)* *=* *0.725; 95% confidence interval (CI): 0.665–0.801] and when there were two children within a family (OR* *=* *0.389; 95% CI: 0.158–0.959). Other factors, such as ethnic minority (OR* *=* *2.266; 95% CI: 1.025–5.012), employment as farm workers (OR* *=* *6.793; 95% CI: 2.203–20.946), and whether unemployed or migrant workers (OR* *=* *7.044 95% CI: 2.491–19.922), were also observed to be risk factors for pediatric burns (Table 5).

**Table 5:** The results of multivariable regression analyses of pediatric burns.

	*β*	SE	Wald	*p*-value	OR	95% CI for OR	
						Lower	Upper
Age	−0.332	0.051	39.723	0.000	0.725	0.655	0.801
Minority	0.818	0.405	4.082	0.043	2.266	1.025	5.012
Number of siblings = 1			4.834	0.089			
Number of siblings = 2	−0.944	0.460	4.205	0.040	0.389	0.158	0.959
Number of siblings ≥ 3	−1.019	0.544	3.507	0.061	0.361	0.124	1.049
Employed			14.592	0.001			
Farmer	1.916	0.575	11.120	0.001	6.793	2.203	20.946
Unemployed or migrant worker	1.952	0.530	13.545	0.000	7.044	2.491	19.922
	0.607^a^	0.615^a^	0.977	0.323	1.836		

^a^ Constant.

### Assessment of goodness-of-fit of the model

**Figure 2:**
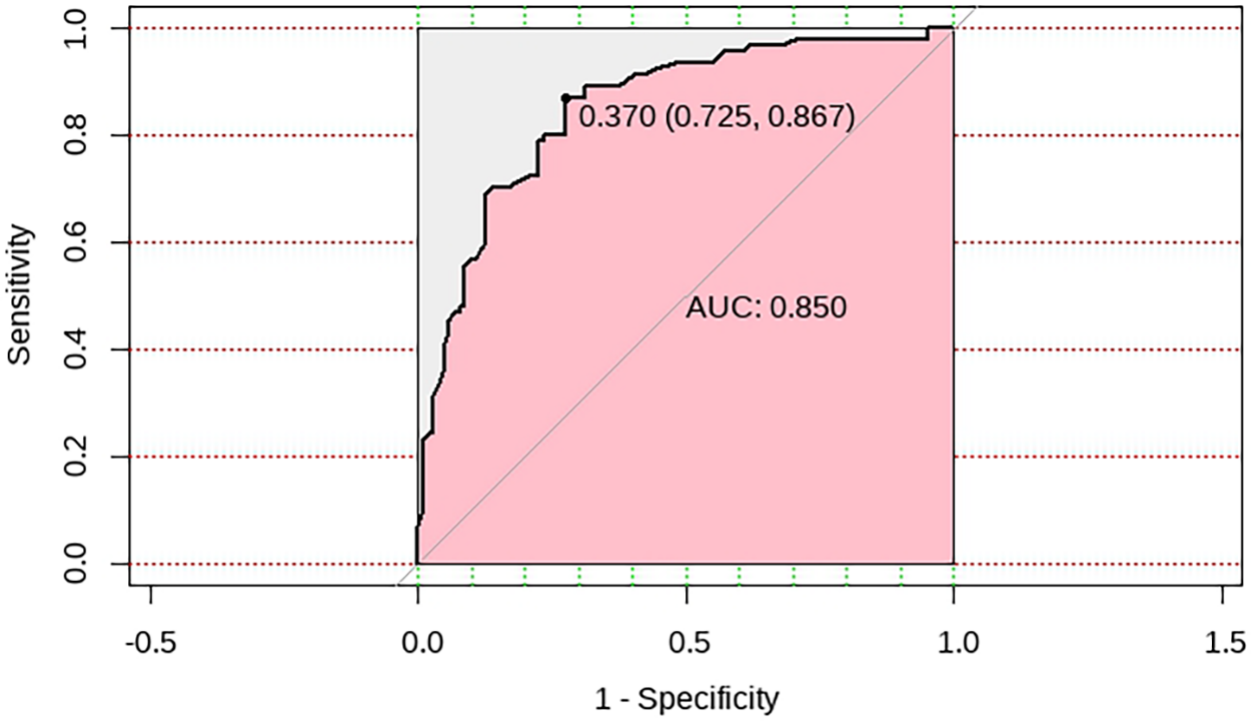
An ROC curve analysis of the risk factors in pediatric burns.

**Figure 3:**
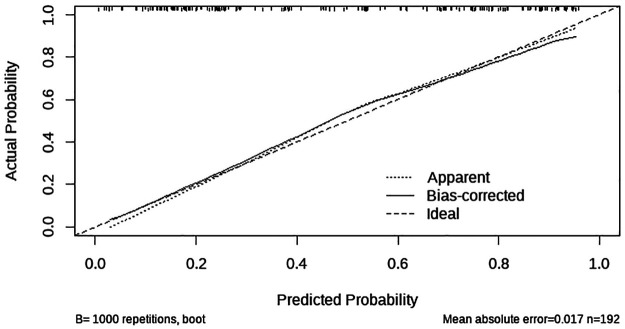
A calibration plot of a multivariate nomogram of risk factors in pediatric burns.

The Hosmer–Lemeshow test was used to assess the goodness-of-fit of the logistic regression model and it was found to be *p* = 0.905 > 0.05. The AUC was 0.850 (95% CI: 0.795–0.905) (Figure 2). For the ICC, a C-index of 0.850 and mean absolute error = 0.017 after 1,000 repetitions was obtained (Figure 3).

### Nomogram analysis of risk factors in pediatric burns

**Figure 4:**
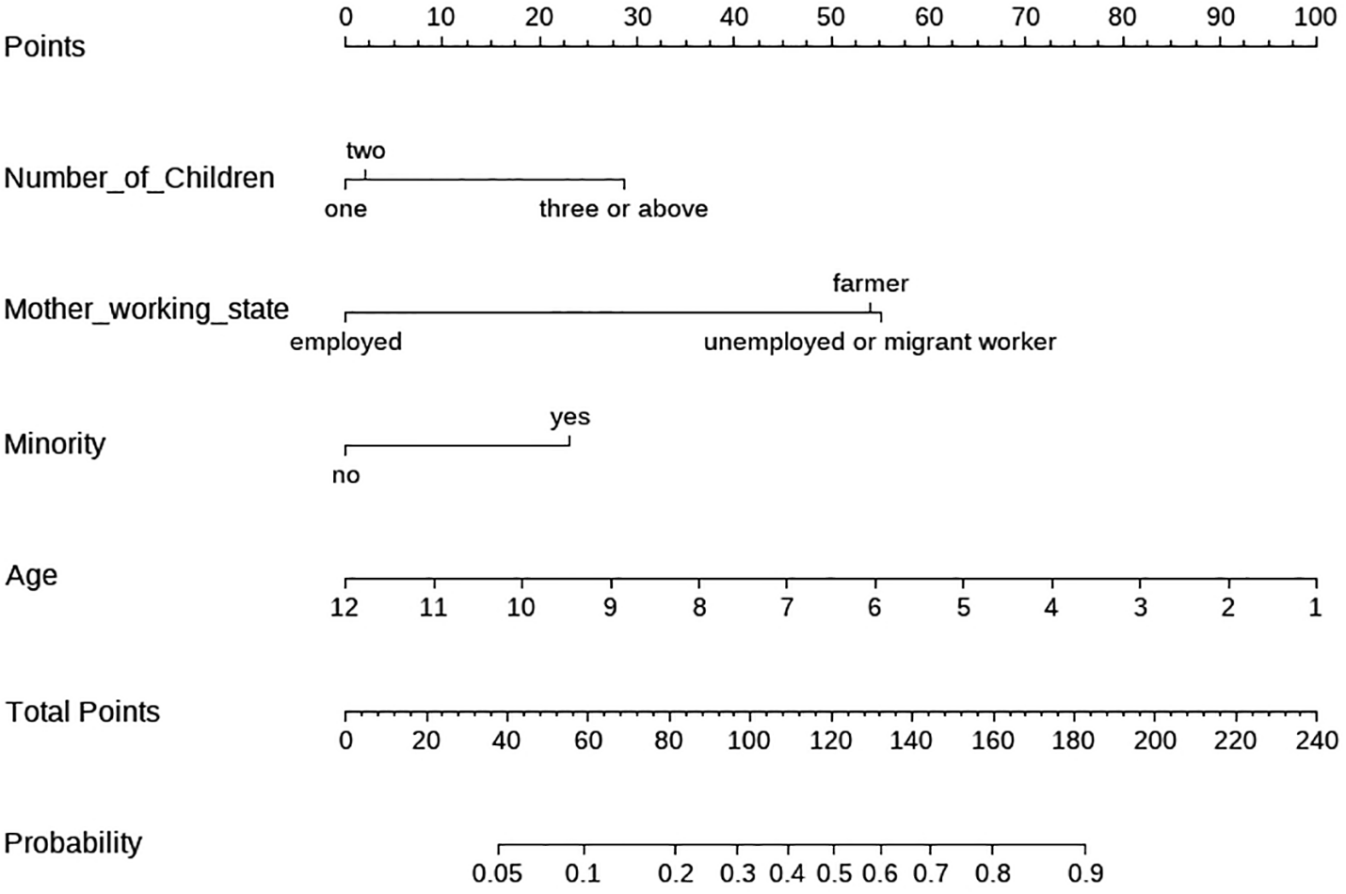
A multifactorial nomogram of risk factors in pediatric burns.

After logistic regression modeling, a nomogram (coefficient plot) was drawn using R Studio 2023.06.0 software to visualize the coefficient of each covariate in the pediatric burns model. From Figure 4, the order in which the covariates contributed to the pediatric burns model is seen to be age > mother’s employment status > number of children in a family > ethnic minority.

## Discussion

The incidence of pediatric burns is unacceptably high globally and efforts should be made to reduce this situation. Wounds associated with burns need to be treated by doctors, and the complex cooperation and treatment process between parents and children as well as the treatment process can often be challenging (5). However, most incidents involving burns are preventable (6). We analyzed the risk factors of pediatric burns using a field questionnaire survey and a case–control study was conducted in an effort to provide guidance as to the best way to reduce the incidents. We found that pediatric burns are invariably due to home accidents, but they are linked to some factors that can increase the risk of their occurrence. In addition, some factors can also play a protective role in the occurrence of pediatric burns (7).

The Han nationality is the most populous ethnic group in China, accounting for more than 90% of the national population (8). However, in Baise, which is a prefecture-level city under the jurisdiction of Guangxi Zhuang Autonomous Region of the People's Republic of China, the Han population accounts for only 16.47% of the total. Several other ethnic minorities account for 83.53% of the population (9, 10). Of the 192 children in this study, 67 (34.90%) were Han and 125 (65.10%) were from other ethnic minorities. The burns group consisted of 54 boys and 36 girls with a boy:girl ratio of 1.5:1. A retrospective, single-center study by Tolouei et al. of children (aged <18 years) admitted to the burns center between 2011 and 2021 showed a similar ratio, with 1,777 boys and 1,174 girls (2). In the study by Han et al., a boy:girl ratio of 1.4:1 was observed in 5,569 pediatric patients in the Burn Research Center, Zhengzhou City between 2013 and 2019 (3).

With respect to our demographic data, we found that the urban population accounted for 25 of the 192 (13.02%) cases, of which there were 12 of 90 (13.33%) burns cases. These results are also in line with the government data, which show that the Baise region is not as economically developed compared with other regions of China, with a low proportion of urbanization and a high proportion of rural population in Baise region (10). Our data in assessing the socio-demographic conditions of pediatric burn wounds were similar to those of Asena et al. (5).

Among the seven covariates we considered, the univariate logistic regression analyses showed that age, the number of children in a family, ethnic minority, and the mother’s employment status were risk factors for pediatric burns. Our results are different from those of another study that found that risk factors such as urban population, gender, and mother’s education level were not statistically significant (11).

Regarding the multivariate regression analysis, age was found to be a protective factor for pediatric burns in our model, and the risk of pediatric burns gradually decreased with increasing age of the children. Age has also been shown to be an influential factor in several other studies. A univariate binary logistic regression analysis confirmed that age was strongly associated with pediatric burns (*p *<* *0.000). A study from the Netherlands looking at severe burn incidents in children aged under 5 years showed that the median age was 18 months (75th percentile: 26), boys: 17 months (75th percentile: 24) and girls: 19 months (75th percentile: 30) (6). Alnababtah et al. (11) and Othman and Kendrick (12) also observed an over-representation of male patients at 13–24 months. The proportion of children <4 years (57/90, 63.33%) in this study was similar to that in other studies. The prevalence of burns increased significantly in children aged 0–4 years when compared to other age groups (13). This may be because younger children tend to be more impulsive, curious, and lack self-awareness, putting them at greater risk of accidental burns exposure (13, 14).

In our study, we found evidence for a decrease in the odds of childhood burn injury for every year increase in age. This result was obtained after adjusting for the confounding factors of ethnicity, number of children, and the mother’s employment status. Mehta et al. analyzed the potential risk factors for childhood burn injury in a district in Ghana and also showed a decrease in the odds of childhood burn injury for every year increase in age (15). Such a conclusion is similar to that of our study. In addition, they also showed that for children in rural Ghana (aged <5 years), having an older child in the family was a protective factor (16). In the study by Han et al., which focused on central China and reported on 5,569 hospitalized children aged 0–14 years, a clear decreasing trend of incidence of pediatric burns with age was shown (3). The reasons given for the gradual decrease in the incidence of burns in children were not only the gradual physical and psychological maturity of children, but their increased ability to identify dangerous objects as well as the formation of different types of educational means and preventive measures (11, 14).

Childhood injury is a major cause of preventable death and disability globally (15). Burns are one of the most common traumatic injuries around the world, and children are usually one of the vulnerable groups (17). In this study, a univariate regression analysis showed that ethnic minority children were more likely to have burn injuries compared with other children. A multivariate regression analysis showed that ethnic minority children contributed to our model after adjusting for age, number of children in a family, and mother’s employment status. Children from families with low socioeconomic status and those that identify as racial or ethnic minorities have consistently been shown to have higher rates of burn injuries globally (18).

In a study by Jeffries et al., the relative risk for injuries requiring hospitalization was calculated for children of Black, Hispanic, and Other races and ethnicities compared with White children (19). Another study cited that childhood burns were associated with young and single-parent families, lower educational level of parents, and lack of safety measures (such as smoke detectors); the influence of racial factors, especially minority children, was over-represented among burn patients worldwide (18). Both these studies emphasized the influence of ethnic minorities on the incidence of pediatric burns. Therefore, it is very important to raise social awareness of burn prevention and fire safety measures (20). It is worth noting that different regions exhibit variations in lifestyle and cooking/heating practices, leading to regional differences. Burn occurrences can be affected by factors such as environmental temperatures and recreational activities. In Switzerland, for instance, burn injuries resulting from fireworks and flames on National Day can sometimes surpass those that occur on New Year's Eve (21). In China, the peak period for children’s burn injuries also occurs in January and February, probably because of New Year's Day and the Spring Festival celebrations (22).

The effect of siblings on the incidence of childhood burns is not entirely consistent. A study by Shah et al. in 2013 showed that the older the sibling was, the higher the odds of scald injury (23). van Zoonen et al. showed that the first-born child was over-represented when compared to second- or third-born children (6). Mehta et al. showed that having older children in the home among children aged* *<* *18 years was protective against injury (15). In addition, another study about household injuries showed that among children aged <5 years, having older children in the household had protective effects (16). These results were slightly different from ours in that the second sibling was protective.

A systematic review by Padalko et al. assessed the relationship between complex social factors and the incidence of pediatric burns. These included factors such as family dysfunction, maternal adolescent pregnancy, newcomers (refugees, asylum seekers, and newcomers), education, mental health, housing, and economic status. Of these, low income, low parental education, behavioral disorders, and rural living were associated with increased social complexity factors that were linked to the risk of burns and which decreased with the mothers’ age (1). Other studies by van Rijn et al. (24), Petridou et al. (25), Delgado et al. (26), Shah et al. (23), and Othman and Kendrick (12) have indicated that low income, poor living standards, mother's low socioeconomic status, deprivation, and minor housing/crowding were associated with child burn injuries.

In this study, we used collinearity to evaluate the performance of our diagnostics used (27, 28). We also used the Hosmer–Lemeshow test (29), AUC (30), and ICC (31, 32) to assess the goodness-of-fit of the logistic regression model. After logistic regression modeling, we used the R language nomogram to visualize the coefficients of each covariate found in the model (33–35).

The present study has some limitations. In this questionnaire survey, the urban/non-urban household registration for analysis data was 25/167 (15%) cases, and we assigned the mothers’ education to high school and above (57 cases) and junior high school and below (135 cases). This may be due to the economic, education, customs, and habits of underdeveloped minority areas; the level of education of mothers in the Baise area was not very high and the urban population ratio was low. Despite the limitations and selection bias of our results, our study is one of the few reports on risk factors in underdeveloped ethnic minority areas in China, and this study provides some valuable insights of risk factors in underdeveloped ethnic minorities in many regions worldwide. In the multivariate regression analysis of the risk variables of pediatric burns, the number of cases was relatively small, and this failed to reach the 1:1 sample size of the case/control group, causing poor statistical power and inevitable confounding bias in the statistical analysis process. We only assessed seven influencing factors in this study and could not comprehensively assess all the possible factors of burns in children. Therefore, future studies on this subject will require longer study time, representative large sample data, and more in-depth studies for more rigorous statistical analysis to be conducted.

Our case–control study used a logistic regression analysis of influencing factors for pediatric burns by using a questionnaire survey. Age was found to be a protective factor for pediatric burns, and minority, mother’s employment status, and number of children in a family were risk factors. The contribution to pediatric burns were age > mother’s employment status > number of children > minority. This study will raise awareness of the specific risk factors associated with pediatric burns and will help government officials to direct their preventive approach accordingly.

## Data Availability

The original contributions presented in the study are included in the article/Supplementary Material, further inquiries can be directed to the corresponding authors.
